# Improving access to antiretrovirals in China: economic analyses of dolutegravir in HIV-1 patients

**DOI:** 10.1186/s12962-019-0195-2

**Published:** 2019-12-05

**Authors:** Yogesh Suresh Punekar, Na Guo, Gabriel Tremblay, James Piercy, Tim Holbrook, Benjamin Young

**Affiliations:** 1 ViiV Healthcare, 980 Great West Road, Brentford, Middlesex, TW8 9GS UK; 2 GlaxoSmithKline, Shanghai, China; 3 Purple Squirrel Economics, New York, USA; 4 Adelphi Real World, Bollington, UK; 5 IAPAC, Washington, DC USA

**Keywords:** Cost effectiveness, Dolutegravir, China, Treatment naive, Economic analyses

## Abstract

**Background:**

The World Health Organisation recommended dolutegravir (DTG)-based antiretroviral therapy (ART) regimens are available but not reimbursed through the public reimbursement system in China. The objective of this analysis was to evaluate the cost-effectiveness of DTG (DTG + TDF/3TC) compared to efavirenz (EFV + TDF/3TC) in treatment-naive and ritonavir-boosted lopinavir (LPV/r + TDF/3TC) in first-line ART failure HIV-1-infected patients in China.

**Methods:**

A dynamic Markov model comprising of 5 response states and 6 CD4+ count-based health states was used. Efficacy, estimated as probability of virologic suppression (HIV RNA < 50 copies/mL) at 48 weeks, was obtained from a published network meta-analysis for ART-naive patients and from the DAWNING study for patients failing first-line ART. Baseline cohort characteristics were informed using DTG phase 3 studies and the DAWNING study data, respectively. Health state utilities were derived from DTG phase 3 studies. A 5-year cost-effectiveness analyses was conducted using the societal perspective. Outcomes were quality-adjusted-life-years (QALYs), life-years (LYs), incremental cost per QALYs (ICER).

**Results:**

The viral suppression rates for DTG + TDF/3TC were higher than EFV + TDF/3TC (75.3% vs 64.0%) in treatment-naive and LPV/r + TDF/3TC (74.8% vs 58.4%) in first-line ART failure patients. This resulted in higher QALYs for DTG + TDF/3TC in treatment-naive (4.232 vs 4.227) and first-line failure settings (4.224 vs 4.221). Total discounted cost for DTG + TDF/3TC patients (RMB 219.259 in treatment-naive and RMB 238,746 in first-line failures) were lower than comparators (EFV + TDF/3TC:RMB 221,605; LPV/r + TDF/3TC:RMB 244,364), thereby DTG dominated in both settings. Probabilistic sensitivity analyses indicated the probability of DTG + TDF/3TC being cost effective was 98.2% in treatment-naive setting and 100% in first-line failure setting at a willingness to pay threshold of RMB 100,000/QALY.

**Conclusions:**

With lower costs, higher response rates and higher QALYs, DTG + TDF/3TC can be considered as a cost-effective alternative for treatment naive and first-line failure patients in China.

## Key messages

- Dolutegravir is highly efficacious antiretroviral therapy recommended as first line treatment in patients living with HIV in most treatment guidelines but is not reimbursed on National Free IADS Antiretroviral Drug List in China.
- Our analyses show dolutegravir to be cost effective compared to efavirenz (EFV) in treatment naïve patients living with HIV (PLHIV) and compared to ritonavir boosted lopinavir (LPV/r) in first line failure PLHIV.
- This provides strong rationale for the adoption of WHO-recommended DTG into first- and second-line HIV treatment regimens in China and may help achieve 90-90-90 ambition set out by Chinese health ministry.

## Introduction

The HIV epidemic in China is characterised by low national prevalence of 0.037% with some regions and some sub-populations reporting higher prevalence [1]. The number of newly diagnosed people living with HIV (PLHIV) continues to increase with 45,000 new cases per year and 758,600 prevalent cases in 2017 [2]. More than 80% of these individuals were receiving antiretroviral therapy (ARTs) in 2017 and this percentage is expected to increase with the government at various levels increasing funds to prevent and effectively manage HIV [1]. The mortality rate among PLHIV is also decreasing with 30,000 reported deaths in 2016 compared to 54,000 in 2009, thus making HIV infection a chronic condition with ageing patients. The government spending on HIV has steadily increased from US$ 139 million in 2006 to US$ 978 million in 2014 before dropping to US$ 843 million in 2015 [2]. Despite more than 90% of funding coming from domestic sources, Chinese government needs to make significant further progress in effective management of HIV. A study [3] conducted in Shandong Province reported 60%, 42% and 15% of all PLHIV being diagnosed, treated and virologically suppressed, respectively. If HIV funding remains curtailed and PLHIV continue to increase, access to effective and cost saving ART will become ever more important in China’s ambition to achieve the UNAIDS 90-90-90 and 95-95-95 targets by 2020 and 2025, respectively.

Policy makers in China have implemented 2015 World Health Organization (WHO) guidelines for HIV treatment which recommend ARTs for all PLHIV irrespective of their CD4 cell counts [4]. The ARTs recommended by WHO in 2013 and further endorsed in 2016 [5], also were subsequently made available through being listed on the National Free AIDS Antiretroviral Drug List. The current landscape of fully reimbursed ART in China include three core agents: two non-nucleoside reverse transcriptase inhibitors (NNRTI) efavirenz (EFV) and nevirapine (NVP), and a single protease inhibitor (PI), ritonavir boosted lopinavir (LPV/r); along with several nucleoside reverse transcriptase inhibitors (NRTI). Combinations of these ART, although effective, have significant potential tolerability issues and have been shown to be associated with long-term toxicities [6]. The process of uptake of newer, more efficacious ARTs has been slow with no integrase strand-transfer inhibitors (INSTIs) being fully or partially reimbursed through government reimbursement funding. This included dolutegravir (DTG) which is a first line treatment recommended by WHO as an alternative option in 2015 and a preferred option in 2018 [4, 7].

DTG is a 2nd generation INSTI that received regulatory approval in China in 2015. DTG has demonstrated superiority to EFV and ritonavir-boosted darunavir (DRV/r) and, non-inferiority to raltegravir (RAL) in its phase 3 clinical program in treatment-naïve HIV-1 infected individuals [8–10]. Two previous network meta-analyses also supported these findings with DTG demonstrating superiority to EFV on efficacy and rates of discontinuations [11, 12]. Authors concluded that some alternative first-line therapies recommended in the 2015 WHO treatment guidelines such as DTG, were superior to WHO-preferred EFV-based ART. Furthermore, in a recent randomised controlled trial in resource constrained settings, DTG has demonstrated superiority to (LPV/r) in HIV-1 patients failing first line ART [13]. Thus, with the demonstrated clinical superiority of DTG in first- and second-line ART regimens, the objective of this analysis was to assess its cost-effectiveness in PLHIV in China.

## Materials and methods

### Model design

A previously published cost-effectiveness model was adapted to estimate the clinical outcomes and costs of DTG + 2NRTIs compared with EFV + 2NRTIs in treatment-naive and compared to LPV/r + 2NRTIs in first-line failure PLHIV. The model framework was a dynamic Markov model and has been described in detail previously [14]. The analyses were performed using a societal perspective, thus including indirect costs and effects.

**Fig. 1:**
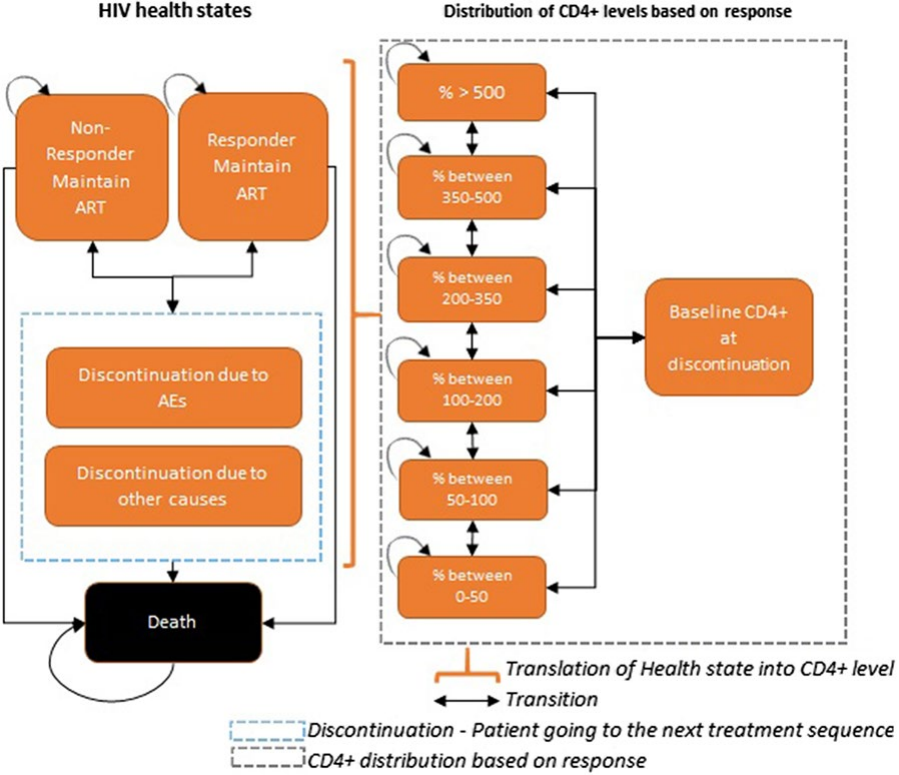
Model figure

In summary, patients entering the model were distributed into one of the six CD4+ based health states depending on their baseline CD4+ level (Fig. 1). Based on their response to treatment, they were stratified in one of the five response states; responders maintaining ARVs, non-responders maintaining ARVs, discontinuations due to AEs, discontinuation due to other causes and death. This resulted in a change in CD4+ level which was a function of the treatment used, the time on treatment and the responder status. Patients responding to treatment improved their CD4+ counts whilst those discontinuing their treatment returned to their baseline CD4+ levels. In addition, the time on treatment was modelled with a linear increase in CD4+ levels until patients reached the trial efficacy at weeks 48 and 96, after which the CD4+ levels were expected to be maintained as long as patients remained on their regimen.

Two separate cost effectiveness analyses were performed, one in treatment-naive patients and the other in individuals experiencing first-line treatment failure.

#### Treatment-naive setting

In the treatment naive analyses, the baseline characteristics of patients entering the model were obtained from DTG phase 3 trials in treatment-naive HIV-1 infected patients [8–10] and further analysed in the NMA by Patel and colleagues [11]. Our analysis compared DTG added to tenofovir/lamivudine (DTG + TDF/3TC) with EFV added to TDF/3TC (EFV + TDF/3TC). Patients discontinuing first-line treatment were assumed to switch to LPV/r + TDF/3TC, the most widely used second-line treatment in China. Treatment efficacy, defined as viral suppression (HIV-1 RNA < 50 copies/ml) leading to increase in CD4+ count, was estimated based on a published network meta-analysis [11]. In this meta-analysis, authors included 31 phase 3/4 randomised controlled trials including 17,000 patients to estimate relative efficacy and safety of DTG compared to guideline-recommended third agents. Bayesian fixed-effect network meta-analyses, adjusted for the NRTI combination, was used to model 48-week viral suppression and changes in the CD4+ cells in people receiving DTG or other core agent comparators. The overall results demonstrated DTG to be comparable to other integrase inhibitors and superior to all other 3rd agents including EFV. The study also reported no significant efficacy difference between all the available ART backbones.

#### First-line failure setting

For the analysis of first-line failures patients, the baseline patient characteristics were obtained from DAWNING study which compared DTG and LPV/r in combination with multiple backbones in HIV-1 patients failing first-line treatment [13]. Treatment efficacy estimates up to 48 weeks were obtained from DAWNING and were used to compare DTG + TDF/3TC with LPV/r + TDF/3TC. In the model, patients discontinuing DTG + TDF/3TC switched to LPV/r + TDF/3TC and vice versa.

Several assumptions and parameters were common to analyses in treatment-naive and first-line failure patients. The CD4+ changes beyond 48 weeks were based on pooled patient level data of all the DTG trials [8–10]. Furthermore, the model assumed the same CD4+ cell count increase rate after 22 months for those suppressed on any regimen, although CD4+ cell count increase differed between treatment regimens. This assumption is consistent with the findings from a US observational study which showed little CD4+ cell increase after 4 years after ART initiation [15]. We assumed that 96 weeks after failing the last ART, patients will experience CD4+ cell count decline, as reported by Mauskopf and colleagues between viral load [16] and CD4+ cell count data from the multicenter AIDS cohort study [15]. The transition probabilities used in the model are displayed in Table 1.

**Table 1:** Model transition probabilities [30]

	DTG + TDF/3TC	EFV + TDF/3TC^a^	LPV/r + TDF/3TC^b^
Virology suppression			
First 11 months	16.3%^a^/15.1%^b^	12.4%	10.4%
Month 12–22	0.2%	0.1%	0.1%
After month 22	0.0%	0.0%	0.0%
Discontinuation due to failure (first line)			
First 11 months	0.46%^a^/0.17%^b^	0.86%	0.60%
Month 12–22	0.10%^a^/0.05%^b^	0.08%	0.04%
After month 22	0.05%^a^/0.02%^b^	0.06%	0.04%
Discontinuation with other cause (first line)			
First 11 months	0.55%^a^/0.63%^b^	0.62%	0.91%
Month 12–22	0.12%^a^/0.18%^b^	0.06%	0.06%
After month 22	0.06%^a^/0.08%^b^	0.04%	0.06%
Discontinuation (second line plus)			
First 11 months	1.03%	1.54%	1.54%
Month 12–22	0.23%	0.10%	0.10%
After month 22	0.10%	0.10%	0.10%
CD4+ increase			
First 11 months	20.29	16.42	15.46
Month 12–22	2.51	0.56	0.56
After month 22	− 2.32	− 2.32	− 2.32

^a^Treatment-naïve setting

^b^First-line failure setting

#### Safety and tolerability

Patients experiencing treatment-related adverse events (AE) were assumed to experience a QoL decrement and additional costs associated with that event. Those experiencing grade 2–4 AEs were assumed to switch to the next treatment. The likelihood of patients experiencing AEs was obtained from the pooled data of DTG phase 3 trials [8–10] in treatment-naive analyses and from DAWNING study in first-line failure analyses [13]. Discontinuations due to AEs were assumed to occur in the first month of treatment (using discontinuation rate at week 48). No a priori relationship was assumed between AEs such that a patient could potentially experience several AEs simultaneously. In such instances, the costs and utility decrements were assumed to be additive.

#### Comorbidities

HIV related comorbidities were assumed to impact utilities and costs. The risk of developing cardiovascular disease (CVD), a common comorbidity among HIV patients, was estimated in each model cycle based on published literature [17]. The other comorbidity considered in the model was the risk of developing an opportunistic infection (OI). Five types of OIs including bacterial, fungal, protozoal, viral, and other infections were included. The risk of occurrence of OIs was dependent on history of that specific OI, patient’s CD4+ cell count, and the time on and response status of treatment. This risk was estimated from a published source [18].

#### Mortality

Mortality included death due to HIV, acute OIs, CVD or natural causes. HIV mortality was estimated based on CD4+ cell count and history of any OI [19]. Acute OI mortality was estimated based on frequency and severity of acute OIs observed in relation to CD4+ counts [15]. All-cause mortality was obtained from life tables in China [20]. Mortality rates used in the model are presented in Appendix Table 4.

### Utilities

Health state level utilities were derived from phase 3 studies of DTG trials and are displayed in Table 2 [8–10]. Disutilities associated with acute OIs and CVD were obtained from studies by Paltiel and Franks, respectively [21, 22]. Disutilities were also applied for grade 2–4 AEs for the duration of the AE [23].

**Table 2:** Health state based utilities and costs used in the model

Health states	> 500	350–500	200–350	100–200	50–100	0–50
Utilities	0.896	0.899	0.886	0.861	0.843	0.822
Costs (RMB)						
Outpatient care	1998	2356	2356	3072	4891	4891
Inpatient care	787	1019	1017	2317	9197	9075
AOI	27	17	18	84	84	201
OI prophylaxis	0	0	0	1	1	2
Cardiovascular event	11	14	17	17	17	17
Indirect costs	0	327	219	1274	1274	1.274

### Costs

#### ART related costs

ART costs were obtained from literature and are presented in Renminbi (RMB) [24]. Generic prices of ARVs were used wherever available. TDF/3TC is the most commonly used, generically available backbone with a monthly cost of RMB 92.39. EFV is the most commonly prescribed 3rd agent among HIV patients initiating treatment and monthly cost of generic EFV (RMB 76.84) was used in the analysis. LPV/r was only available as a branded product with a monthly cost of RMB 343.78. It was assumed that DTG, if reimbursed, would be priced on parity to LPV/r.

#### HIV management costs

HIV management costs included testing and switching costs [25], physician outpatient costs [25], HIV hospitalisation [26] and costs of treatment and prophylaxis of OIs [26].

Costs associated with management of frequent comorbidities such as cardiovascular disease also were included. These were estimated using the prevalence of CVD among HIV patients in China and their associated costs [27]. All the costs were further stratified by health states using monthly resource utilisation estimated by d’Armino and colleagues [18].

#### Indirect costs

Indirect costs comprising of productivity losses were included in the model. Productivity losses were estimated from literature [28] and costed using Chinese data.

In the base case, model time horizon was 5 years based on the mean duration of first-line ART in China [29]. Both direct and indirect healthcare costs were included and, costs and benefits were discounted at 2.3% per year (Inflation index China). All costs are reported in RMB and inflated to 2017 values.

### Economic analyses

The incremental efficacy in quality adjusted life years (QALYs) and incremental costs of DTG + TDF/3TC was compared with EFV + TDF/3TC in treatment-naive patients and with LPV/r + TDF/3TC in first-line failure patients. The incremental cost effectiveness ratio (ICER) was reported as cost per responder and cost per QALY. Model robustness was assessed with multiple one-way deterministic sensitivity analyses by varying key parameters through plausible ranges of ± 20%. Parameters varied were CD4+ efficacy, AE prevalence, costs and utilities. Cost effectiveness estimates over model time horizons ranging from 1 year to patient lifetime were explored. Probabilistic sensitivity analysis (PSA) was used to estimate the impact of parameter uncertainties.

## Results

### Treatment-naive setting

**Fig. 2:**
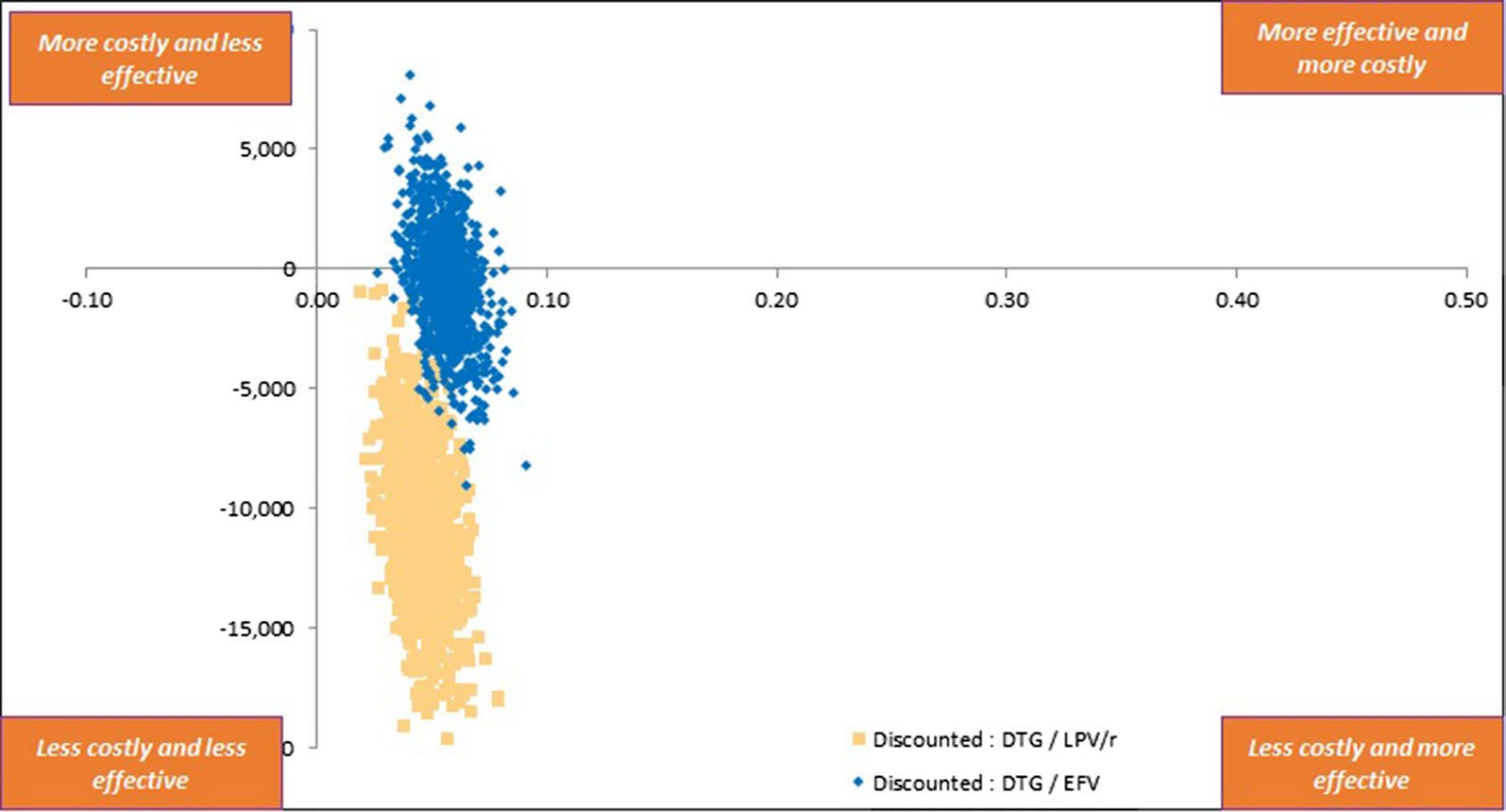
Cost effectiveness plane—DTG + TDF/3TC vs EFV + TDF/3TC (treatment-naïve) and DTG + TDF/3TC vs LPV/r + TDF/3TC (first-line failures)

The base case model predicted 75.3% patients on DTG + TDF/3TC and 64.0% patients on EFV + TDF/3TC to achieve and maintain virologic suppression. The mean duration of response per patient over 5 years was 41.7 months for DTG + TDF/3TC and 36.7 months for EFV + TDF/3TC. This resulted in 4.232 QALYs per patient on DTG + TDF/3TC and 4.227 QALYs per patient on EFV + TDF/3TC. The discounted and half-cycle corrected total costs of HIV treatment per patient over 5 years was RMB 219,259 for DTG + TDF/3TC and RMB 221,605 for EFV + TDF/3TC. The incremental cost effectiveness analyses showed DTG + TDF/3TC resulting in 0.006 incremental QALYs with cost savings of RMB 467 compared to EFV + TDF/3TC, thus dominating EFV based regimen (Table 3). One-way sensitivity analyses suggested that CD4 + improvements due to DTG + TDF/3TC or EFV + TDF/3TC and price of DTG + TDF/3TC were key drivers of cost effectiveness results. Probabilistic sensitivity analyses showed that at a willingness to pay threshold of RMB 100,000, the probability of DTG + TDF/3TC being cost effective was 98.2% (Fig. 2).

**Table 3:** Costs, outcomes and ICERs compared to DTG + ABC/3TC

	Treatment naïve		First line failures	
	DTG + TDF/3TC	EFV + TDF/3TC	DTG + TDF/3TC	LPV/r + TDF/3TC
Efficacy				
Responders (%)	75.3	64.0	74.8	58.4
Months of response	41.7	36.7	41.4	33.7
Life years	4.728	4.728	4.728	4.728
QALYs	4.232	4.227	4.224	4.221
Costs (in RMB)				
Total	219,259	221,605	238,746	244,364
ART	24,744	11,958	24,744	24,744
Routine care	173,930	184,364	187,859	192,237
AEs and other events	13,297	13,357	13,092	13,182
Indirect costs	7289	11,926	13,052	14,202
ICERs (including indirect costs)				
Costs/responder	DTG dominates		DTG dominates	
Costs/QALY	DTG dominates		DTG dominates	
ICERs (excluding indirect costs)				
Costs/responder	DTG dominates		DTG dominates	
Costs/QALY	DTG dominates		DTG dominates

### First-line failure setting

**Fig. 3:**
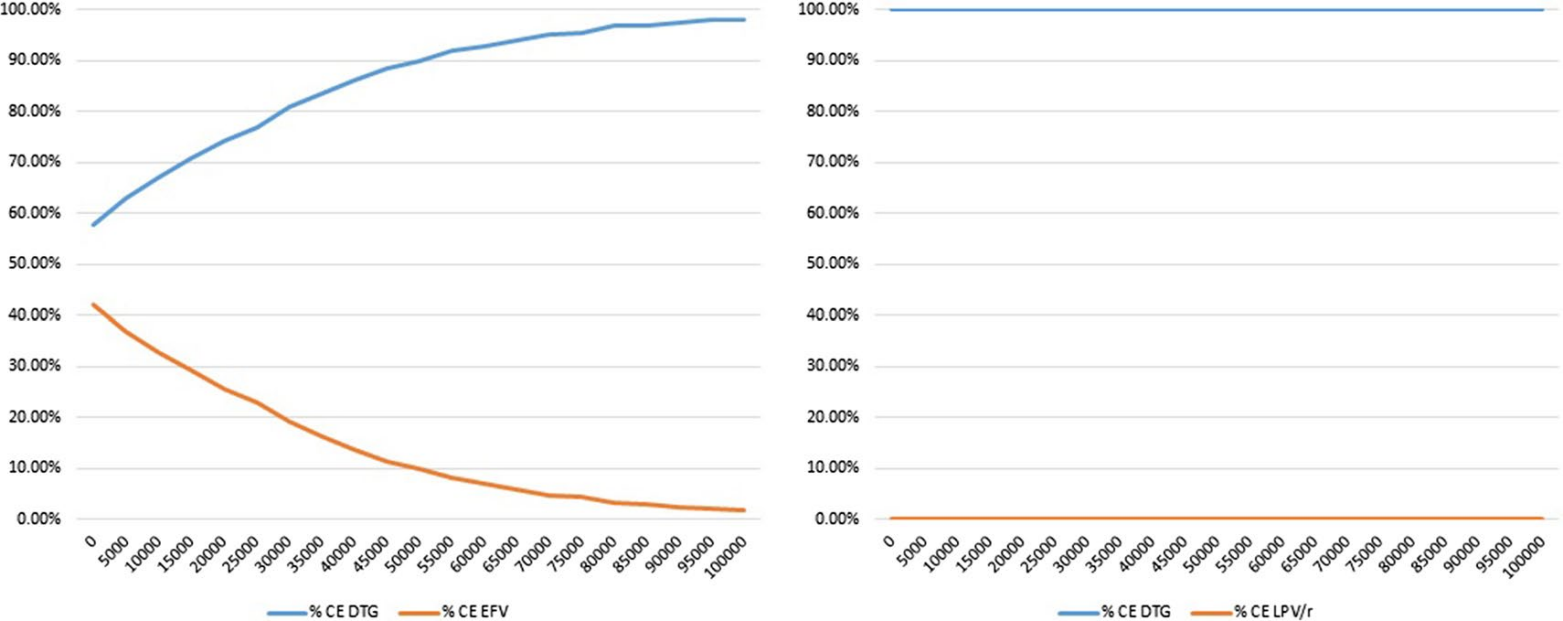
Cost effectiveness plane and cost effectiveness acceptability curve—DTG + TDF/3TC vs LPV/r + TDF/3TC

In the base case analysis, the model predicted 74.8% of DTG + TDF/3TC patients and 58.4% LPV/r + TDF/3TC patients achieving and maintaining viral suppression. This resulted in mean response duration of 41.4 months and 33.7 months, and mean QALYs of 4.224 and 4.221, for the two treatment alternatives over 5 years, respectively. The total costs for DTG + TDF/3TC were lower compared to LPV/r + TDF/3TC (RMB 238,746 vs RMB 244,364) resulting in DTG based regimen dominating LPV/r based regimen. One-way sensitivity analyses identified utility estimate of the CD4 +>500 health state and prices of DTG + TDF/3TC and LPV/r + TDF/3TC as key drivers of the results. At a willingness to pay threshold of RMB 100,000, DTG + TDF/3TC had 100% probability of being cost-effective (Fig. 3).

## Discussion

Our study compared cost effectiveness of DTG + TDF/3TC with EFV + TDF/3TC in treatment naive HIV-1 infected patients and with LPV/r + TDF/3TC in patients with first-line ART failure in China. Based on the NMA and the patient level data, the model predicted that DTG-based regimens will achieve higher viral suppression, higher CD4+ counts and consequently higher QALYs compared to currently reimbursed, most widely used treatments in both settings. This finding is consistent with the clinical profile of DTG which has shown superiority to both these alternatives in respective patient populations in head-to-head randomised clinical trials. The total direct and indirect costs of HIV management were lower for the DTG based regimen, thereby dominating the comparators. Sensitivity analyses suggested that the results were plausible and likely stable. Our results are also aligned with multiple published economic analyses which have concluded that DTG-based regimens are dominant or cost effective compared to EFV or LPV/r based regimen in multiple countries and settings [30–34].

In our analyses, we used viral suppression and CD4 + change estimates derived using published NMA by Patel and colleagues. We preferred this over the head-to-head study comparing DTG + (abacavir (ABC)/3TC) and EFV + (TDF/emtricitabine (FTC)) [10] as the SINGLE study used different NRTI combinations which could contribute to differential efficacy of full regimens. The NMA instead reported core agent efficacies controlling for the effect of the backbone which was more appropriate for our analyses. A more recent NMA was available but not incorporated into our modeling since it did not report CD4+ comparisons essential for QALY calculations in our model [12]. Both NMAs reported consistent results for all ARTs in treatment naïve setting. We therefore believe that our results will be similar to those reported here, if repeated using Kanters analyses.

Our analyses resulted in ICERs that can be considered conservative. Due to the limited information available in the NMA [30], we attributed no additional viral suppression or QALY benefit to DTG beyond 2 years despite one DTG clinical trial demonstrating continued efficacy up to 3 years [14]. Further, several studies have demonstrated that if better viral suppression leads to improvements in CD4 + counts, then it may also lead to reduction in mortality [10]. Although all the interventions compared in our analyses have demonstrated improvements in CD4 + counts among treatment naïve HIV-1 patients, none has shown a conclusive benefit in mortality. We therefore excluded reduction in mortality attributable to CD4 + improvements from our analyses resulting in a potential underestimate of DTG efficacy.

Our analysis has limitations. We estimated ART efficacy from randomized clinical trials; it’s possible that real-world results, or those in China may differ. ART outcomes will also be affected by levels of adherence and persistence, prevalence of AEs and baseline CVD risk among HIV patients in clinical practice. In our analysis, we did not include these parameters due to lack availability of robust estimates specific to HIV patients in China. Some of the resource use estimates as well as clinical estimates such as risk of OIs were derived from older studies which may over or under estimate the current HIV resources in China. Incremental implementation costs or feasibility favouring either DTG or comparators were also not modelled.

Recent 2018 WHO HIV treatment guidelines [7] recommend DTG + TDF + 3TC as the preferred initial ART for adults, adolescents and children and DTG as a preferred component for second-line ART for individuals experiencing failure of first-line NNRTI-based ART. This cost effectiveness analysis provides strong additional support for the adoption of WHO-recommended DTG into first- and second-line HIV treatment regimens in China.

## Conclusion

HIV-1 infected treatment naïve and first line failure patients achieved higher response rates and higher QALYs at lower costs, when treated with DTG + TDF/3TC compared to currently available ARV regimens. Inclusion of dolutegravir in the National Free AIDS Antiretroviral Drug List at an optimal price may offer healthcare professional and policy makers, an additional option in tackling the growing burden of HIV in China.

## Data Availability

Important data likely to influence clinical practice and healthcare policy are included in this published article and its supplementary information files. Any additional datasets used and/or analysed during the current study are available from the corresponding author on reasonable request.

## Appendix

See Table 4.

**Table 4:** CD4+ monthly mortality rate

	> 500 (%)	350–500 (%)	200–350 (%)	100–200 (%)	50–100 (%)	0–50 (%)
Normal mortality	0.0002					
Adjusted natural mortality	− 0.0005					
HIV mortality (raw)	0.7000	1.2000	1.8000	8.0000	8.0000	8.0000
HIV mortality (monthly adjustment)	0.0585	0.1006	0.1513	0.6924	0.6924	0.6924
Average HIV mortality	0.1850					
AOI mortality rate (monthly)						
m	0.041	1.020	4.476	0.960	3.100	3.700
Fungal	0.106	0.331	0.348	0.135	0.591	1.123
Protozoal	0.003	0.009	0.504	0.067	0.140	0.270
Viral	0.006	0.013	0.696	0.214	0.523	1.857
Other OI	0.047	0.087	0.224	0.716	2.460	3.940
AOI final mortality (monthly)	0.009	0.065	0.364	0.105	0.326	0.530
Average AOI rate	0.181					
Used in the model	0.067	0.165	0.515	0.796	1.018	1.222
Monthly CD4+ mortality rate	0.067	0.165	0.515	0.796	1.018	1.222

Regarding the correction for all-cause mortality, a standardised mortality ratio by CD4+ cell count was applied as derived by Lewden et al. [35]
